# Building Primary-School Children’s Resilience through a Web-Based Interactive Learning Environment: Quasi-Experimental Pre-Post Study

**DOI:** 10.2196/27958

**Published:** 2021-06-09

**Authors:** Iolie Nicolaidou, Evi Stavrou, Georgia Leonidou

**Affiliations:** 1 Department of Communication and Internet Studies Cyprus University of Technology Limassol Cyprus; 2 Dyslexia Centers Limassol Limassol Cyprus

**Keywords:** COVID-19, interactive learning environment, internet-based cognitive behavioral therapy, parents, prevention intervention, primary school children, psychological resilience, teachers

## Abstract

**Background:**

Resilience is a person’s mental ability to deal with challenging situations adaptively and is a crucial stress management skill. Psychological resilience and finding ways to cope in crises is a highly relevant topic considering the COVID-19 pandemic, which enforced quarantine, social distancing measures, and school closures worldwide. Parents and children are currently living with increased stress due to COVID-19. We need to respond with immediate ways to strengthen children’s resilience. Internet-based cognitive behavioral therapy interventions for children's stress management overcome accessibility issues such as the inability to visit mental health experts owing to COVID-19 movement restrictions. An interactive learning environment was created, based on the preventive program “Friends,” to overcome accessibility issues associated with delivering cognitive behavioral therapy–based interventions in formal and informal education settings.

**Objective:**

This study aimed to examine the effectiveness of a web-based learning environment on resilience in (1) reducing anxiety symptoms and (2) increasing emotion recognition and recognition of stress management techniques among 9-10-year-old children. We also aimed to evaluate the learning environment’s usability.

**Methods:**

A quasi-experimental pretest-posttest control group design was used. In total, 20 fourth graders in the experimental group interacted with the learning environment over 6 weekly 80-minute sessions. Further, 21 fourth graders constituted the control group. The main data sources were (1) a psychometric tool to measure children’s anxiety symptoms, namely the Greek translation of the original Spence Children’s Anxiety Scale, (2) 3 open-ended questions assessing emotion recognition and recognition of stress management techniques, and (3) the System Usability Scale to measure the usability of the learning environment.

**Results:**

In both groups, there was a small but nonsignificant postintervention reduction in reported anxiety symptoms, except for obsessive-compulsive disorder symptoms in the experimental group. A paired samples *t* test revealed that students’ reported symptom scores of obsessive-compulsive disorder significantly decreased from 1.06 (SD 0.68) to 0.76 (SD 0.61) (*t*_19_= 5.16; *P*=.01). The experimental group revealed a significant increase in emotion recognition (*t*_19_=–6.99; *P*<.001), identification of somatic symptoms of stress (*t*_19_=–7.31; *P*<.001), and identification of stress management techniques (*t*_19_=–6.85; *P*<.001). The learning environment received a satisfactory usability score. The raw average system usability score was 76.75 (SD 8.28), which is in the 80th percentile rank and corresponds to grade B.

**Conclusions:**

This study shows that interactive learning environments might deliver resilience interventions in an accessible and cost-effective manner in formal education, potentially even in distance-learning conditions owing to the COVID-19 pandemic. Interactive learning environments on resilience are also valuable tools for parents who can use them with their children at home, for informal learning, using mobile devices. As such, they could be a promising first-step, low-intensity intervention that children and the youth can easily access.

## Introduction

Resilience refers to a person’s mental ability to adaptively deal with challenging situations and is a key skill for dealing with challenges in life [1], which typically cause stress, which, if left untreated, may escalate to anxiety. According to Ye [2], stress is the body’s physical and mental response to the awareness of major changes or threats. Anxiety is one of the most common childhood mental health conditions [3,4]. A lifetime prevalence as high as 30% prior to 18 years of age has been reported in adolescents in the United States from the general population, with a median age of onset of 6 years [5,6]. Furthermore, the prevalence of subclinical anxiety has been estimated at a much larger proportion, approaching 40% in children [5].

According to Ye [2], emotional states and clinical symptoms are influenced by the COVID-19 pandemic, requiring psychological assistance and care. Anxiety is a common emotional reaction during the current pandemic [7]. Specifically, “school-aged children may be more nervous and scared, and repeatedly ask parents about the situation of the pandemic. Adolescents may have worries, irritability, and tantrums” [2]. Parents and children are living with increased stress owing to COVID-19 [8]. According to Dalton et al [9], “children are exposed to large amounts of information and high levels of stress and anxiety in the adults around them. Simultaneously, children are experiencing substantial changes to their daily routine and social infrastructure, which ordinarily foster resilience to challenging events” [9].

Stress is a problem for children and adolescents as it can have a negative emotional and social effect on children’s mental health and quality of life both in school and at home. There is an overall consensus that stress should be addressed early on, through prevention interventions, before it escalates to anxiety. Prevention interventions can equip children with skills that will protect them from future mental disorders and can therefore help reduce the need for future therapeutic interventions.

The effectiveness of conventional face-to-face instructional interventions for preventing anxiety and for building mental resilience has been shown through systematic reviews that have examined the efficacy and effectiveness of school-based prevention programs for anxiety [10]. Most of these studies were based on cognitive behavioral therapy (CBT) and have shown that most of the evaluated programs were effective in reducing the symptoms of anxiety in children and adolescents. Prevention programs have been carried out in different settings, including formal education and informal education settings [11], and typically followed rigorous methodologies, including randomized controlled trials [12]. Cognitive behavioral therapy has therefore been demonstrated to be effective and is a well-established treatment for children and adolescents with anxiety disorders [3,4,13]. Meta-analyses have concluded that approximately 60% of children following CBT treatment typically recover from the anxiety disorder that causes most interference (ie, the primary anxiety disorder) [4].

One of the most widely used and recognized CBT-based programs for preventing anxiety in children is called “Friends” [14]; it involves 10 sessions, and it was based on a program formerly called the “Coping Cat.” The program was evaluated and yielded positive outcomes in Hong Kong [14], Canada [15], and other countries, and it was found to be effective in developing children’s skills in managing stress. The program focuses on 3 areas: body symptoms, cognitive procedures, and management skills. Children learn relaxation and breathing techniques as ways for stress management, and they also learn how to convert negative thoughts into realistic perceptions and positive thoughts [16].

The effectiveness of the “Friends” program has been demonstrated through several studies that reported a significant reduction in stress among children who participated in the program when these were compared with control groups [17-19]. More favorable outcomes were obtained when health professionals, as opposed to educators, were involved in running the sessions [20]. There have been studies that have not shown the superiority of this program when compared to a control condition, as stress symptoms in children equally declined in both conditions [21]. Other studies have reported that the program was more beneficial for low-risk children than for high-risk children [22]. Waldron et al [23] reviewed 8 different studies that evaluated the program and reported that 5 of 8 were effective. Simultaneously, Johnstone et al [24] compared prevention intervention programs that include a large number of sessions, such as the Friends program, to short-term programs as part of their meta-analysis. They found that the former is generally more effective in teaching children how to reduce anxiety and manage stress.

There are various accessibility issues for conventional face-to-face interventions for anxiety, such as the high cost of treatment by specialized mental health experts [25], other barriers to the receipt of treatment, such as accessibility, stigma, and privacy [26] inequalities in health, emotional or practical obstacles [27], or more recently the inability to visit a mental health expert owing to movement restrictions related to the COVID-19 pandemic in various countries, including the United States [28] and China [7]. Technology can eliminate some of these obstacles. One relatively new and increasingly popular approach of increasing access to treatment is the use of web-based intervention programs [29]. For example, internet-based CBT (iCBT) for children and adolescents is a persuasive system that combines three major components to therapy: therapeutic content, technological features, and interactions between the user and the program, intended to reduce users’ anxiety symptoms [30]. According to a scoping review by Ashford et al [29], the advantage of web-based approaches is the accessibility, affordability, and anonymity of potentially evidence-based treatment. In their meta-analysis including internet-based interventions for children, the youth, and young adults with anxiety, Ye et al [31] reported that these interventions could be effective in reducing the severity of symptoms in the youth and can further be considered comparable with conventional programs that have the same goal. Several studies that were based on CBT and targeted children [32] have reported positive results with respect to the effectiveness of internet-based interventions for children aged 7-13 years [33], 9-14 years [34], and younger children aged 4-11 years [35]. For example, March et al [26] demonstrated the efficacy, feasibility, and acceptability of a web-based, publicly available self-help iCBT for children and adolescents with anxiety by assessing program adherence and satisfaction and significant changes in anxiety.

The target of studies focusing on how technology facilitates the delivery of CBT interventions in the treatment of psychological disorders is mostly adults. Fewer data exist for computer-based (standalone, self-help) and computer-assisted (in combination with face-to-face therapy or therapist-guided) programs for the youth [36]. Another problem is that relatively few web-based interventions on the world wide web have provided evidence of the intervention’s efficacy, if we consider the treatment of anxiety as an example [29], and these are not necessarily appropriate for children, as the majority of them mostly target an adult population. From the large number of web-based programs available on the internet and the large number of apps that can be downloaded on mobile phones and tablets [37], only a few have been systematically tested and have published data on feasibility, acceptability, efficacy, and effectiveness [38]. For example, none of the mobile phone apps in previous studies [35,36] on eHealth interventions for anxiety management targeting young children and adolescents had published data derived from trials that examined their efficacy. Lastly, Stiles-Shields et al [39] reviewed CBT-informed behavioral intervention technologies for youth with anxiety and reported that prevention interventions receive lesser attention than therapeutic interventions, and Tozzi et al [36] confirmed this finding in their review.

Children spend a significant part of their day at school. Therefore, schools are an important setting for promoting psychological resilience in the youth [40]. The lack of easily accessible, empirically validated prevention interventions addressing school children’s needs for emotional resilience have necessitated this study. This need is pressing, as the world is currently struggling to curb the influences of the COVID-19 pandemic, and as there are indications that the quarantine and social distancing policies will have long-term impacts on children’s mental health [2]. According to Ye [2], innovative digital solutions and informatics tools are needed more than ever to mitigate these negative consequences on children. According to March et al [26], if effective, iCBT programs could be a promising first-step, low-intensity intervention that children and the youth can easily access. The present study attempted to examine whether a CBT-based prevention intervention that aims to build resilience in young children can be effective when enacted in formal education through a web-based, interactive learning environment.

## Methods

### Research Questions

This study focused on designing and evaluating an interactive learning environment, which was enacted in formal education, to examine its potential to deliver resilience interventions in an effective and accessible manner. The research questions of the study were the following:

1. To what extent is the interactive learning environment for resilience effective in reducing 9-10-year-old children’s anxiety symptoms?
2. To what extent is the interactive learning environment for resilience effective in increasing 9-10-year-old children’s skills in identifying emotions and stress symptoms and in recalling stress management techniques?
3. How do 9-10-year-old children evaluate the usability of the interactive learning environment for resilience?

### Interactive Learning Environment for Resilience

For the purpose of the study, an interactive learning environment to support resilience was designed and developed in accordance with the structure and recommendations of Psyllou [41], who based her work on the conventional CBT program “Friends.” The protagonists in the learning environment were 6 distinct 9-year-old characters who acted as peer models for children and showed them how to manage their anxiety. The learning environment included the following: (1) narrations that children could listen to on headphones, (2) children’s individual and anonymously provided written reflections on personal struggles, which were made possible through a comment feature that was only visible to the administrator (ES), to respect children’s privacy, and (3) interactive quizzes, which provided instant feedback.

The web-based environment involved a series of interactive activities structured in six 80-minute sessions. In session 1, children were introduced to the 4 primary emotions (happiness, anger, sadness, and fear) and individually responded to web-based questions asking them to identify when and how specific people felt each emotion. Web-based questions also prompted them to think about times when they (or others) felt the same emotion. The final activity of session 1 required children to draw their self-portrait to show what they look like when they feel these 4 emotions.

In session 2, children were introduced to stress and anxiety, through short stories they could listen to from each of the 6 protagonists. The protagonists showed them that everyone is stressed out by different situations by sharing their own anxieties. The children were then prompted to answer web-based questions to identify an area or situation that was stressful for them with which they would like to learn how to cope.

In session 3, the protagonists explained the relationship between thoughts and emotions and demonstrated how children can change their negative thoughts to positive ones. The children could listen to brief examples derived from the protagonists’ daily life in which they experienced something that made them anxious. They then responded to web-based questions that asked them to identify a time during the previous week when they felt happy and one during which they felt anxious and describe their thoughts and emotions in each case. They were then asked to listen to a short story and respond to web-based questions to identify positive and negative thoughts that the protagonists might have had.

In session 4, children had more practice in identifying positive and negative thoughts through a web-based quiz that provided instant feedback. In the same session, they learned how they can ignore the negative thoughts and retain the positive ones through several examples of short stories narrated by the protagonists. During the last activity in session 4, the children applied what they have learned in a situation that was stressful for them by responding to web-based questions to describe this situation and by identifying a positive thought relevant to that situation.

In session 5, the protagonists taught children how to manage stressful situations and demonstrated how they could design action plans to gradually face their fears by breaking them down into manageable small pieces. Children first listened to one of the protagonists’ narration of how she overcame the anxiety of reading in front of the whole class by focusing on 1 step at a time: the protagonist started out by reading a short text alone at home in front of a mirror (step 1), one of the protagonist’s parents (step 2), the protagonist’s whole family (step 3), one of the protagonist’s friends (step 4), a group of friends (step 5), and eventually the whole class (step 6). Students responded to web-based questions to identify a stressful situation for them and to break it down to 6 smaller steps. In Session 6, the children were reminded of everything they have learned, including the acronym of the word “friends,” which helps children remember the steps to follow upon feeling anxious [42]: F=“feeling worried?” R=“relax and feel good,” I=“inner thoughts (changing negative thoughts to positive ones),” E=“explore action plans,” N=“nice work, reward yourself,” D=“don’t forget to practice,” and S=“stay calm.”

Several key persuasive systems’ design features were used in the interactive learning environment, including “simulation with a social role,” “similarity,” and “social learning” [30]. For example, “simulation with a social role” was incorporated in instances where the 6 animated characters narrated their experiences while simultaneously illustrating concepts of the program. “Social learning” was incorporated when these animated characters provided suggested solutions or worked through their problems to serve as peer models for children and to demonstrate ways to solve real-life problems. “Similarity” was incorporated because the examples and activities provided in the learning environment were specific to target 9-10-year-old children and their typical everyday stressors (similarity), which included the anxiety of speaking in front of the whole class or in front of a bigger audience, and anxiety related to taking school tests.

### Study Design

The study followed a quasi-experimental pretest-posttest control group design. A primary school with access to a computer laboratory was chosen through convenience sampling to participate in the study. Children individually interacted with the web-based learning environment (1 child:1 computer), using headphones. The learning environment was enacted under the responsibility of author ES (who also designed the learning environment) in close collaboration with author GL (a licensed psychologist), who was present in all sessions. The decision to include a psychologist was based on research findings from the evaluation of the conventional program “Friends,” in which more favorable outcomes were found when health professionals, as opposed to educators, were involved in conducting the sessions [20]. However, as the intervention took place primarily on the internet, the guidance provided by either the psychologist or author ES to children while interacting with the learning environment was minimal and focused on technical issues of navigation rather than psychological issues.

Based on previous studies [20,25,41], it was decided that the most appropriate age group for the intervention is 9-10 years, which corresponds to children who are in the fourth grade of primary school. There were 2 fourth grade classes in the school selected in this study. One class was randomly considered the experimental group, while the other served as the control group.

### Participants

In total, 20 fourth-grade primary school students (11 boys and 9 girls) were included in the experimental group and used the web-based learning environment over six 80-minute lessons between November 2018 and March 2019. In addition, 21 fourth-grade primary school students (10 boys, 11 girls) were included in the control group and did not receive formal instruction on resilience, emotion recognition, or stress management.

### Ethical Considerations

The study protocol was approved by the Center for Educational Research and Evaluation of the country in reference (proposal reference# 7.15.01.25.8.1/9), and it was evaluated by the ethics committee of the university in reference (proposal submission number 54) prior to the conductance of the study. The study adhered to the ethical standards of the American Psychological Association and General Data Protection Regulation guidelines. Our study adhered to all legal requirements of the country where it was conducted. All participants were informed in writing about the study’s objective, and the students’ parents signed the consent forms for their children to participate in the study voluntarily.

### Data Sources and Data Analysis

Data sources were the following: (1) the Greek translation of the psychometric tool Spence Children’s Anxiety Scale (SCAS) to measure children's anxiety symptoms [43-45], (2) 3 open-ended questions to assess the ability to recognize emotions and anxiety symptoms and to recall ways of managing stress, and (3) the System Usability Scale (SUS) to measure the usability of the learning environment. These 3 data sources were used to answer research questions 1, 2, and 3, respectively.

The psychometric tool SCAS [43,44] is a child self-reported tool that consists of 45 questions designed to measure symptoms related to separation anxiety (questions 5, 8, 12, 15, 16, and 44), social phobia (questions 1, 6, 7, 9, 20, 29, and 35), obsessive-compulsive disorder (OCD; questions 14, 19, 27, 40, 41, and 42), panic agoraphobia (questions 3, 13, 21, 28, 30, 32, 34, 36, 37, and 39), generalized anxiety (questions 2, 18, 23, 25, and 33), and fears of physical injury (questions 1, 3, 4, 20, 22, and 24). The scale consists of 45 statements outlining anxiety symptoms, to which children report with the frequency by which they experience these symptoms by using a 4-point Likert scale (0=“never,” 1=“sometimes,” 2=“often,” and 3=“always”). Six of the 45 statements are positive statements that aim to reduce the negative predisposition toward statements outlining anxiety symptoms. These 6 statements are not typically included in the data analysis. The scale was translated and weighted for the Greek population [45].

To analyze data from the psychometric tool, the Greek translation of the original SCAS for research question 1, the response “never” received a score of 0, the response “sometimes” received a score of 1, the response “often” received a score of 2, and the response “always” received a score of 3. The total score of the symptoms was computed for each of the 6 anxiety disorders examined in this instrument so that the mean score of each disorder could be computed. Descriptive statistics (mean, SD) and inferential statistics (paired samples and independent samples *t* tests) were used for data analysis. An α level of .05 was set a priori for all statistical analyses.

For research question 2, 3 open-ended questions were used to assess the ability to recognize emotions and symptoms of anxiety and to recall ways of managing stress. These were the following: (1) “What are the four main emotions?” (2) “What symptoms do you feel on your body when you are stressed?” and (3) “What can you do to relax when you feel stressed?” The coding sheets for the evaluation of these 3 open-ended questions are provided in Tables 1, 2, and 3.

**Table 1:** Coding sheet for an open-ended question on basic emotion identification.

Points received	Rationale	Example student answer
0	No reference to emotions	“*I don’t know*” [Participant #1, female]
1	Reference to 1 of 4 primary emotions	“*Happiness*” [Participant #10, male]
2	Reference to 2 of 4 primary emotions	“*Happiness, Sadness, Joy*” (joy is not one of the 4 basic emotions) [Participant #6, female]
3	Reference to 3 of 4 primary emotions	“*Happiness, anger, excitement, sadness*” (excitement is not one of the four basic emotions) [Participant #16, female]
4	Reference to the 4 primary emotions	“*Happiness, anger, fear, sadness*” [Participant #3, female]

**Table 2:** Coding sheet for an open-ended question on the identification of somatic symptoms when stressed.

Points received	Rationale	Example student answer
0	No reference of symptoms	“*I don’t have any symptoms*” [Participant #1, female]
1	Reference to 1 symptom	“*Trembling*” [Participant #6, female]
2	Reference to 2 symptoms	“*I have pain in my belly, and I tremble*” [Participant #13, male]
3	Reference to ≥3 symptoms	“*I am sweating, I have a headache, and I tremble*” [Participant #3, female]

**Table 3:** Coding sheet for an open-ended question on the identification of stress management techniques.

Points received	Rationale	Example student answer
0	No reference to stress management techniques	“*I do nothing*” [Participant #1, female]
1	Reference to 1 stress management technique	“*I lie down*” [Participant #3, female]
2	Reference to 2 stress management techniques	“*I can sleep and watch TV*” [Participant #19, male]
3	Reference to ≥3 stress management techniques	“*I close my eyes, watch TV, I use my Playstation*” [Participant #13, male]

Descriptive statistics (mean, SD) and inferential statistical tests (paired samples and independent samples *t* tests) were used for data analysis for answering research question 2.

For research question 3, the learning environment’s usability was measured with the SUS [46]. The SUS was selected because (1) it is a highly robust and versatile tool for usability professionals [47] and (2) it allows for the comparison of similar systems. The scale included the following ten items, with responses graded on a 5-point Likert scale ranging from “completely disagree” to “completely agree”: (1) “I think that I would like to use this learning environment frequently,” (2) “I found the learning environment unnecessarily complex,” (3) “I thought the learning environment was easy to use,” (4) “I think that I would need help from my parents or siblings to be able to use this learning environment,” (5) “I found the various functions in the learning environment were well integrated,” (6) “I thought there was too much inconsistency in the learning environment,” (7) “I would imagine that most children my age would learn to use this learning environment very quickly,” (8) “I found the learning environment very cumbersome to use,” (9) “I felt very confident using the learning environment,” and (10) “I needed to learn a lot of things before I could get going with this learning environment.”

For data analysis, the procedure for calculating usability evaluation scores proposed by Brooke et al [46] was followed. Specifically, for odd-numbered items, 1 was subtracted from the user response. For even-numbered items, the user responses were subtracted from 5. This procedure scored all values on a scale of 0 to 4 (4 being the most positive response). The converted responses for each user were summed, and the total was multiplied by 2.5. This converted the range of possible values of 0-100 instead of 0-40. An average SUS score was calculated for all participants. The SUS score was then converted into a percentile rank and a letter grade from A to F in accordance with the norms proposed by Sauro [48,49].

## Results

### Research Question 1

All 41 learners were pretested with regard to their level of experience of anxiety disorders. Group equivalence was first established. There were no significant differences between the 2 classes when an independent samples *t* test was performed (*t*_38_=0.083; *P*=.93) to compare the pretest scores of students’ separation anxiety for the intervention group (mean score 1.00, SD 0.69) and the control group (mean score 0.99, SD 0.58). The same finding was obtained for all anxiety disorders, as shown in Table 4, indicating that the 2 groups were equivalent before the intervention.

After establishing group equivalence, experimental students’ pre- and postintervention scores for anxiety disorders were compared. A paired samples *t* test revealed that students’ reported symptom scores of OCD significantly decreased from 1.06 (SD 0.68) to 0.76 (SD 0.61) (*t*_19_= 5.16; *P*=.01). A post hoc power analysis was performed using G*Power3 [50] where 1-b was computed as a function of α (set at .05), the population effect size parameter for a medium effect size (Cohen d=.05), and the sample size used in this study (n=20 for the experimental group). The power thus calculated was 0.695. Reported scores for the symptoms of separation anxiety, social phobia, fears of physical injury, and generalized anxiety decreased slightly but nonsignificantly from before to after the intervention (Table 4). Panic agoraphobia symptoms were, however, slightly but nonsignificantly increased.

Furthermore, we compared the scores for students’ anxiety disorders before and after the intervention for the control group. In total, the scores for 4 of 6 disorders (separation anxiety, OCD, panic agoraphobia, and fears of physical injury) were slightly but nonsignificantly reduced from before to after the intervention (Table 4).

**Table 4:** Anxiety disorders before and after the intervention for the experimental and control groups.

Anxiety disorders	Experimental group (n=20)		Control group (n=21)		Comparison of pretest scores	
	Preintervention, mean (SD)	Postintervention, mean (SD)	Preintervention, mean (SD)	Postintervention, mean (SD)	*t* value	*P* value
Separation anxiety	1.00 (0.69)	0.79 (0.62)	0.99 (0.58)	0.93 (0.58)	0.083^a^	.93
Social phobia	0.97 (0.57)	0.75 (0.47)	0.87 (0.54)	0.99 (0.67)	0.550^b^	.58
Obsessive-compulsive disorder	1.06 (0.68)	0.76^c^ (0.61)	0.97 (0.60)	0.83 (0.59)	0.213^d^	.83
Panic agoraphobia	0.39 (0.48)	0.45 (0.51)	0.49 (0.41)	0.45 (0.48)	–0.721^e^	.62
Generalized anxiety	0.78 (0.39)	0.77 (0.58)	0.90 (0.61)	0.87 (0.57)	0.203^a^	.84
Fears of physical injury	0.83 (0.66)	0.60 (0.57)	0.79 (0.59)	0.82 (0.52)	–0.760^a^	.45

^a^df=38.

^b^df=37.

^c^*P*=.01.

^d^df=36.

^e^df=39.

### Research Question 2

All 41 learners were pretested with regard to their level of knowledge of the 4 basic emotions, their ability to identify somatic stress symptoms, and their ability to identify ways to manage their stress. As shown in Table 5, students in the control group initially outperformed those in the experimental group in the pretest score. We observed a significant difference between the 2 classes when an independent samples *t* test was performed (*t*_39_=0.005; *P*=.005) to compare the students’ total pretest score for the experimental (mean 3.35, SD 1.79) and control (mean 5.05, SD 1.83) groups.

However, in general, the performance score of the students in the control group was lower in the posttest (mean 4.71, SD 1.52) rather than the pretest (mean 5.05, SD 1.83) conditions. For the 3 areas that were examined, the performance of the students in the control group remained the same after the intervention, as was the case for the identification of the 4 basic emotions (mean 2.86, SD 1.35) or it was lower, as was the case for the identification of somatic symptoms of stress and stress management techniques.

On the contrary, the performance of the students in the experimental group increased from 3.35 (SD 1.79) of 10 in the pretest condition to 7.65 (SD 0.88) in the posttest condition. This increase was significant (*t*_19_=–10.46; *P*<.001) and a large effect was observed (Cohen d=1.88; 95% CI 1.136-2.625) [51].

As shown in Table 5, a significant increase was also observed for students in the experimental group with regard to the identification of basic emotions after the intervention, where a large effect was observed (Cohen d=1.22; 95% CI 0.546-1.897) [51]. Significant, large effects were also observed for the identification of somatic symptoms of stress (Cohen d=1.56; 95% CI 0.853-2.269) and the identification of stress management techniques (Cohen d=1.248; 95% CI 0.57-2.925).

**Table 5:** Identification of basic emotions, somatic stress symptoms, and stress management techniques before and after the intervention for the experimental and control groups.

Anxiety disorders	Experimental group (n=20)			Control group (n=21)	
	Preintervention, mean (SD)	Postintervention, mean (SD)	*t* value^a^	Preintervention, mean (SD)	Postintervention, mean (SD)
Identification of the 4 basic emotions	1.55 (1.05)	3.35^b^ (0.05)	–6.99	2.86 (1.35)	2.86 (1.01)
Identification of somatic symptoms of stress	0.70 (0.66)	2.15^b^ (0.13)	–7.31	1.05 (0.74)	1.00 (0.77)
Identification of stress management technique	1.05 (0.69)	2.15^b^ (0.67)	–6.85	1.14 (0.73)	0.86 (0.73)
Total score	3.35 (1.79)	7.65^b^ (0.88)	–10.46	5.05 (1.83)	4.71 (1.52)

^a^Paired samples *t* test; df=19.

^b^*P*<.001.

### Research Question 3

The average SUS scores reported for the 20 children in the experimental group were calculated to answer research question 3. The learning environment received a satisfactory usability score. The raw average SUS score was 76.75 (SD 8.28), which was in the 80th percentile rank and corresponded to grade B.

**Figure 1:**
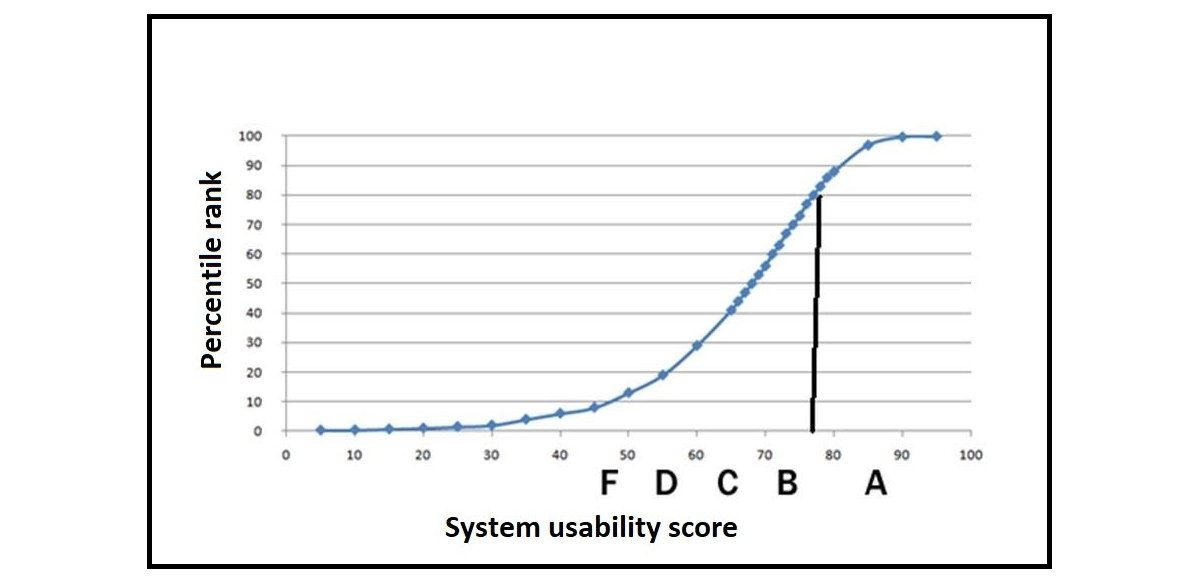
The raw average system usability score of the interactive learning environment on resilience, based on a sample of 20 participants. A, B, C, D, and F are the corresponding letter grades.

Figure 1 shows how the percentile ranks are associated with SUS scores and letter grades [48,49]. This process is similar to “grading on a curve” based on the distribution of all scores. For example, a raw SUS score of 74 converts to a percentile rank of 70. A SUS score of 74 has higher perceived usability than 70% of all products tested [48,49].

## Discussion

This study aimed to examine the effectiveness of a technology-supported learning environment on resilience in reducing anxiety symptoms among 9-10-year-old children and in increasing children’s recognition of emotions and stress management techniques through a quasi-experimental pretest-posttest control group design. The study initially showed that anxiety symptoms of participating students generally manifested at a low level in both the experimental and control groups, as prior to the intervention, all anxiety disorders that were examined, specifically separation anxiety, social phobia, OCD, panic agoraphobia, generalized anxiety, and fear of physical injury received low scores that did not exceed 1 on a scale ranging from 0 (“never”) to 3 (“always”). This finding suggests that students, on average, “sometimes” experience symptoms associated with the aforementioned disorders. Despite low levels of reported anxiety symptoms before the intervention, there was a significant reduction in symptoms associated with OCD after the intervention in the experimental group, which indicates that the learning environment on resilience effectively reduced reported symptoms associated with OCD, especially since the students in the control group did not display a significant reduction in their scores. A comparison of pretest and posttest scores for the experimental group revealed a slight but nonsignificant reduction in the frequency of reported symptoms associated with disorders, such as separation anxiety, social phobia, generalized anxiety, and a fear of physical injury.

Regarding research question 2, the interactive learning environment on resilience was shown to be effective in increasing children’s ability to recognize basic emotions, identify somatic stress symptoms, and recall stress management techniques they can use in real-life settings to alleviate stress. This was evident from a significant increase in their scores for open-ended questions after the intervention both overall and for each specific area examined, as opposed to the control group, whose performance scores did not increase.

Psychological resilience and finding ways to cope during a crisis is a highly relevant topic owing to the recent COVID-19 pandemic, which enforced quarantine and social distancing measures and school closures worldwide. During the pandemic, the youth have faced challenges associated with the loss of face-to-face social interaction [52]. According to Ye [2], psychological crises often cause children to have feelings of abandonment, despair, incapacity, and exhaustion and even raise the risk of suicide. Children with mental illnesses are especially vulnerable during the quarantine and social distancing period. Even though the world has been struggling to curb the influences of the pandemic, quarantine and social distancing policies will have long-term impacts on children [2]. Therefore, according to Ye [2], innovative digital solutions and informatics tools are needed more than ever to mitigate the negative consequences on children. March et al [26] reported that web-based self-help CBT might offer a feasible and acceptable first step for delivering mental health care services to children and adolescents with anxiety. Accordingly, the interactive learning environment on resilience that was described and evaluated in our study is an example of a digital solution toward this direction. As revealed by research question 1, the learning environment was found to be effective in reducing anxiety symptoms of OCD, at least based on children’s self-reports; moreover, the learning environment effectively supported children in identifying stress symptoms and in recalling stress management techniques through a web-based intervention with minimal guidance from a teacher or mental health expert.

Pusey et al [1] recently reported that interactive technologies can deliver effective resilience interventions in an accessible, cost-effective, and flexible manner. Their review included several types of interactive technologies used in resilience interventions, such as serious video games, virtual reality simulations, social robots, and commercial off-the-shelf video games. Their review did not, however, include web-based interactive learning environments. Our study shows that interactive learning environments seem to have the potential to deliver resilience interventions in formal education settings in an accessible and user-friendly manner as well, as revealed by our usability findings. Children in this study were assisted by an adult to a very small extent as they mostly navigated through the learning environment using headphones at their own pace and individually responded to web-based questions. The proposed resilience intervention can potentially be accessible beyond the classroom’s confined environment for providing support to children anywhere and anytime, with a smaller need for support by adults. This would allow for children’s individual, independent use of the intervention at their own pace at school or at home. The web-based learning environment on resilience can be a useful and empirically validated digital resource for parents to use with their children at home or for teachers to use in classes conducted on the internet to focus on their mental health, which can be delivered either synchronously or asynchronously.

### Limitations

The sample of the study was small and random assignment in the 2 conditions was not possible. Convenience sampling was used rather than random sampling, which would have been preferred to increase the generalizability of our findings.

The psychometric instrument that we used was lengthy and included difficult terms. Its completion proved to be challenging for students. Moreover, even though it is a standardized instrument, it relies on the accuracy of young children’s self-reporting of symptoms. It would have been better if the data on students’ reported anxiety symptoms were triangulated with the use of additional qualitative data collection methods, such as parental interviews or teacher interviews. The role of parents and teachers in the program was potentially important, as it is possible that they might have already performed other effective interventions to manage children’s mental health. This contextual information was not collected at the time and is therefore not available to support our understanding of the characteristics of the selected participants.

Overall, the study was based on self-reported quantitative data. The addition of qualitative data in the form of a large number of student interviews would strengthen the study. Only 4 interviews were conducted with students of the experimental group, whose assessments are not included in this study. Longitudinally measuring students’ anxiety symptoms would also significantly add to the study, as it is possible that such interventions may have long-term effects rather than short-term effects, as previous studies have shown [14,23,53].

### Future Directions

Ignoring the immediate and long-term psychological effects of the COVID-19 pandemic would be unconscionable, especially for children and the youth, who account for 42% of our world’s population [9]. With respect to short-term future goals, we therefore urgently need to utilize effective strategies to strengthen teachers and families to respond to the global situation of the pandemic, as suggested by Cluver et al [8]. We need to respond with immediate ways to strengthen children’s resilience, as “COVID-19 is not the first virus to threaten humanity, and it will not be the last” [8]. iCBT for child and adolescent anxiety has demonstrated efficacy in randomized controlled trials, but it has not yet been examined when disseminated as a public health intervention [26]. Effective iCBT programs could be a promising first-step, low-intensity intervention that can be easily accessed by the youth [26]. As a short-term research goal, the proposed intervention can be disseminated to a large sample of students who are currently taking classes through distance-learning while being isolated at home owing to COVID-19 movement restrictions imposed by several countries.

The interactive learning environment on resilience was used with children of the general population who experienced minimal stress at the time of the intervention (2018-2019), and it was shown to reduce 9-10-year-old children’s anxiety symptoms slightly. It might prove to be more valuable when used by high-risk rather than low-risk children. This could be a direction for future studies. As it is currently unknown whether implications from this study can be applied to different age groups, using the learning environment on resilience with children who are younger or older than the children that were recruited in this study could be another direction for future studies.

Engagement with interactive technology and whether users engage with target behaviors outside of the interactive technology in reference is difficult to measure and remains an unresolved challenge [1,35,54,55]. With respect to long-term future goals, studies should aim to follow-up with participants through longitudinal studies and embed evaluation systems that will enable assessing intervention fidelity and adherence to suggested stress management techniques in real-life settings. In this study, children only accessed the interactive learning environment at school. In future studies, especially if the intervention is made accessible among students on their mobile devices outside of school, there might be a way to measure “acceptability, dropout rates, and frequency of use,” as measures that would imply engagement, according to Pusey et al [1].

## Abbreviations

iCBT: internet-based cognitive behavioral therapy
OCD: obsessive-compulsive disorder
SCAS: Spence Children’s Anxiety Scale
SUS: System Usability Scale
